# Targeting Mesenchymal Stromal Cells/Pericytes (MSCs) With Pulsed Electromagnetic Field (PEMF) Has the Potential to Treat Rheumatoid Arthritis

**DOI:** 10.3389/fimmu.2019.00266

**Published:** 2019-03-04

**Authors:** Christina L. Ross, Dennis C. Ang, Graça Almeida-Porada

**Affiliations:** ^1^Wake Forest Institute for Regenerative Medicine, Winston-Salem, NC, United States; ^2^Wake Forest Center for Integrative Medicine, Wake Forest School of Medicine, Winston-Salem, NC, United States; ^3^Department of Rheumatology and Immunology, Wake Forest School of Medicine, Winston-Salem, NC, United States

**Keywords:** pulsed electromagnetic field (PEMF), rheumatoid arthritis (RA), mesenchymal stromal cells/pericytes (MSCs), osteogenesis, chondrogenesis, angiogenesis

## Abstract

Rheumatoid arthritis (RA) is a systemic autoimmune disease characterized by chronic inflammation of synovium (synovitis), with inflammatory/immune cells and resident fibroblast-like synoviocytes (FLS) acting as major players in the pathogenesis of this disease. The resulting inflammatory response poses considerable risks as loss of bone and cartilage progresses, destroying the joint surface, causing joint damage, joint failure, articular dysfunction, and pre-mature death if left untreated. At the cellular level, early changes in RA synovium include inflammatory cell infiltration, synovial hyperplasia, and stimulation of angiogenesis to the site of injury. Different angiogenic factors promote this disease, making the role of anti-angiogenic therapy a focus of RA treatment. To control angiogenesis, mesenchymal stromal cells/pericytes (MSCs) in synovial tissue play a vital role in tissue repair. While recent evidence reports that MSCs found in joint tissues can differentiate to repair damaged tissue, this repair function can be repressed by the inflammatory milieu. Extremely-low frequency pulsed electromagnetic field (PEMF), a biophysical form of stimulation, has an anti-inflammatory effect by causing differentiation of MSCs. PEMF has also been reported to increase the functional activity of MSCs to improve differentiation to chondrocytes and osteocytes. Moreover, PEMF has been demonstrated to accelerate cell differentiation, increase deposition of collagen, and potentially return vascular dysfunction back to homeostasis. The aim of this report is to review the effects of PEMF on MSC modulation of cytokines, growth factors, and angiogenesis, and describe its effect on MSC regeneration of synovial tissue to further understand its potential role in the treatment of RA.

## Introduction

Rheumatoid arthritis (RA) is a systemic autoimmune disease affecting over 1.3 million Americans, and as much as 1% of the population worldwide (1). Although RA predominantly affects large and small joints, it can affect other organs in the body, including those of the cardiovascular, pulmonary, and ophthalmologic systems (2). The pathophysiology of RA includes abnormal activation of blood cells, namely macrophages, T-cells, and B-cells, which produce pro-inflammatory mediators (e.g., cytokines and growth factors) that initiate an inflammatory cascade that leads to joint damage (i.e., bone erosions) and systemic complications (3). Current treatments include corticosteroids, traditional disease-modifying anti-rheumatic drugs (DMARDs), and anti-cytokines (biologics); however, these drugs have adverse effects which can be severe, including osteoporosis, alterations of metabolism, infection, bone marrow suppression, hepatitis, and an increased risk of malignancies (4–6). As the disease progresses, joints are damaged resulting in impaired range of motion, joint deformity, and dysfunction (7). Although the currently approved drugs are known to prevent further joint damage, the effect of these drugs in repairing bone erosions has yet to be demonstrated, and pro-anabolic agents are needed to promote bone formation at the erosion sites (8). Therefore, innovative and safe strategies aimed at both reducing inflammation and promoting tissue regeneration are urgently needed to inhibit the progression of RA.

A promising novel strategy for the treatment of RA is the local or systemic delivery of extremely low frequency pulsed electromagnetic fields (PEMF) to target mesenchymal stromal cells/pericytes (MSCs) to improve their ability to modulate immune responses and repair tissue. PEMF are physical stimuli that affect biological systems through the production of coherent or interfering fields that modify fundamental electromagnetic frequencies generated by living organisms (9, 10). PEMF activate multiple intracellular pathways, including numerous processes and biochemical mechanisms within both the immune and microvascular systems. There are two methods in which PEMF can be applied to biological tissues: capacitive or inductive coupling. In direct capacitive coupling, an electrode must be placed on the tissue (11); however, in non-direct capacitive coupling/inductive coupling, electrodes do not have to be in direct contact with the tissue because the electric field produces a magnetic field that, in turn, produces a current in the conductive tissues of the body (11–13). PEMF therapy is based on Faraday's law, a basic law of electromagnetism that predicts how a magnetic field will interact with an electric circuit to produce an electromotive force known as electromagnetic induction. This law dictates the more charge that is needed, the higher the intensity of the PEMF signal needs to be. This is represented by the equation dB/dT, where B is peak magnetic intensity, T is time, and d is the derivative (or change) in these units. Since the PEMF signal needs to be able to pass deep enough through the tissue to produce healing results, field intensity, frequency, and time of exposure are all important components in the dosimetry. PEMF follows the inverse square law, so it drops off exponentially from the distance of the surface of the coil; therefore, the closest tissue to the coil (applicator) gets the maximum intensity, and furthest tissue from the coil gets the least intensity.

PEMF can alter cell function by triggering the forced vibration of free ions on the surface of the plasma membrane, causing external oscillating field disruptions in the electrochemical balance of transmembrane proteins (ion channels) (9, 14). It has been suggested that PEMF may be propagated and effectively amplified along the entire signal transduction pathway, thereby modifying cell behavior (15–17). Indeed, several studies have reported that PEMF can modulate both cell surface receptor expression/activation, and downstream signal transduction pathways, thereby restoring homeostatic cell functions such as viability, proliferation, differentiation, communication with neighboring cells, and interaction with components of the extracellular matrix (ECM) (18–23).

By modulating the expression of various signaling cascades and cellular information processing networks to potentially restore them to homeostatic (healthy) production levels, PEMF is showing promise as a treatment for autoimmune diseases such as RA (24–27). Changes in the cells' microenvironment are integrated into a survival response by complex signal transduction mechanisms (28). Lipid nanopores forming stable, ion channel conduction pathways in the plasma membrane of cells (29), explain the conduction of ions into the cell from the extracellular space, specifically calcium (Ca^2+^) ion flux (17, 30, 31). It has been postulated that a direct effect of PEMF on phospholipids within the plasma membrane stimulates the production of second messengers, initiating multiple intracellular signal transduction pathways (32–34).

PEMF intensity is dependent upon wave amplitude/field strength measured in units of Tesla (T), or Gauss (10,000 T). In order to deliver a therapeutic PEMF, it is necessary to optimize three important parameters: frequency, intensity, and duration/time of exposure (9). Previous studies have conclusively shown that optimization of the frequency, intensity, and time of exposure is helpful in attaining consistent beneficial results in experimental arthritis in rats (35–37). A 5 Hz frequency, 4 microT (μT) intensity, applied for 90 min to the rat paw was reported to be the optimal dosimetry for lowering edema, and reducing swelling, inflammatory cell infiltration, hyperplasia, and hypertrophy of cells lining the synovial membrane (37). Preliminary studies in humans have also reported that PEMF can reduce chronic joint swelling and pain in patients with RA (25). Further, the beneficial effects of PEMF have been reported to last up to 3 months or longer in human patients with chronic inflammatory/autoimmune disorders (38) with no evidence of adverse effects (39).

## PEMF Modulates RA Tissue Pathogenesis via Modulation of MSCs and FLS

Normal synovium composition consists of a well-organized matrix of fibroblast-like cells (FLS) and macrophage-like cells known as synovial cells or synoviocytes. The joint-lining synovial membrane consists of a layer of macrophage-like (type a) synoviocytes, fibroblast-like synoviocytes (FLS–type b), and mesenchymal stromal cells (MSCs) (40). In RA, the synovium becomes infiltrated by cells of lympho-hematopoietic origin, namely T-helper cells, B cells, and macrophages, which cause synovial hyperplasia and neoangiogenesis (7, 41, 42). The resulting inflammatory response poses considerable risks for joint damage, and articular dysfunction if left untreated (43). Type A synoviocytes are CD163+, CD68+, CD14^+/lo^ cells that localize to the intima and the subintimal layers of the synovial membrane and proliferate in response to inflammatory conditions. Under pathological conditions, Type A (macrophage-like) synoviocytes contribute to cartilage destruction by producing pro-inflammatory cytokines. They originate in the bone marrow, like other mononuclear phagocytes, and are constantly replaced via the circulation. In rheumatoid synovium sections, 80–100% of the synovial lining cells are macrophage-like cells functioning as antigen processing- and antigen-presenting cells to T lymphocytes (44). Type A synoviocytes also induce the formation of osteophytes through the release of transforming growth factor-beta (TGF-β) 3 and bone morphogenetic proteins (BMP)-2 and BMP-4 (45).

FLS, a heterogeneous population of fibroblastic cells, express CD55 and also play a central role in the maintenance of joint inflammation and the destruction of cartilage (8, 46). RA joint pathology is characterized by chronic inflammation of the synovium (synovitis), which causes cartilage and bone erosion between inflammatory/immune cells and resident FLSs (47). Under healthy conditions, these cells contribute to the homeostasis of normal joints by synthesizing extracellular matrix (ECM) molecules and secreting specific components of synovial fluid (48). Synovial Fibroblasts respond to inflammatory cytokines, mainly TNF-α, by producing a large variety of inflammatory mediators along with tissue destruction (49, 50).

MSCs are also shown to be present in various areas of the joint (51). Immunoregulatory function of MSCs can be modulated by proinflammatory cytokines such as IFN-γ, TNF-α, and IL-1α or β (52). Synovial MSCs express CD44, CD90, CD271, and UDPGD, required for hyaluronan synthesis, and possess high chondrogenic potential (53). Synovial MSCs, which when healthy, maintain tissues and facilitate the repair process. While both FLSs and MSCs are part of the synovium, their functional specialization and diversification may be dependent on their positional information and environmental cues (54); however the relationship between MSCs and FLSs remains unclear. MSCs in the synovial lining could be perhaps stem cells interspersed between the FLSs and synovial macrophages. Alternatively, the FLSs could be a stage of differentiation of the MSC lineage, taking on FLS-specific properties, but still maintaining their MSC lineage (54).

While immune cells have been extensively investigated in the pathogenesis of RA, little is known about the *in vivo* functions of FLSs/MSCs in the regulation of immune homeostasis in physiology and their contribution to immune regulation in RA. Under normal conditions, FLSs/MSCs would control the degree of immune responses; however, the inflammatory environmental signals cue inflammatory cells, unsettling the immunomodulatory functions of FLSs/MSCs, damaging the pannus, contributing to chronic disease maintenance and progression (55). Aberrant cross-talk between FLSs/MSCs and immune cells (T-cells, B cells and macrophages) could be a vicious cycle of chronic RA progression (54). This could be due to MSCs ability to express inflammatory mediators such as prostaglandin E_2_ and IL-6. Also enzymatic production of arachidonic acid enhanced in MSCs by TNF-α or IFN-γ have a deleterious effect on immune cells in the RA microenvironment (56). Thus, heterogeneity of MSCs in terms of immune and hematopoietic function can either maintain immune homeostasis or promote RA pathogenesis.

Healthy MSC function has been shown to inhibit inflammatory responses and improve regeneration (57, 58) by: (a) inhibiting inflammatory cell infiltration and inflammatory cytokine release (59); (b) activating regulatory T-cells (Tregs) (60); and (c) influencing the transition from Th1 cells toward Th2 cells (61). MSCs exert their regulatory activities through the release of immunomodulatory molecules such as IL-10, TGF-β, PGE_2_, and indoleamine 2,3-dioxygenase (IDO) (62, 63). In addition, MSCs are able to polarize macrophage differentiation toward the anti-inflammatory M2 phenotype *in vitro* and *in viv*o (64, 65); inhibit T-cell proliferation (61, 66); and induce the formation of Tregs (67, 68). As such, MSCs are an attractive target for immunomodulation, particularly in the treatment of cartilage injuries and diseases such as RA (54), as modulation of resident synovial MSCs could lead to the control of the inflammatory immune response (57) and ultimately decrease the RA-associated angiogenesis processes.

Stimulation of resident MSCs, or other tissue specific cells to improve inflammation and/or tissue regeneration, is a relatively new concept in medicine that could potentially be achieved by the use of PEMF (10, 69–72). PEMF has the potential to prevent aberrant and promote healthy MSC function. PEMF has been shown to induce differentiation of MSCs to promote immunomodulation and improve cartilage and bone regeneration *in vitro* (10) and *in vivo* (73). Stimulation of chondrogenesis *in situ* through PEMF could lead to an increase of cartilage matrix and collagen levels in RA damaged joints (24, 26, 27, 30, 74, 75). In addition, PEMF promotes proliferation of endogenous chondroblasts (73), supports the enhancement of cartilage regeneration (76), and potentiates MSCs' anti-inflammatory responses. In RA, PEMF also upregulates adenosine receptors to increase anti-inflammatory effects on both chondrocytes and FLS and reduces levels of enzymes produced by FLS and osteoclasts that lead to bone destruction (24, 27, 77) (Table 1).

**Table 1:** Frequency Specific Effects of PEMF on cells and tissues associated with RA.

**Authors**	**Frequency (Hz)**	**Field strength (mT)**	**Time of exposure**	**Outcome**
Chen et al. (78)	15	2	8 h/day	Increased cartilaginous matrix deposition and enhanced chondrogenic gene expression in SOX-9, COL II, and aggrecan in MSCs
De Mattei et al. (79)	75	2.3	At 1, 6, 9, and 18 h for 3 and 6 days	Increased proliferation of human articular chondrocytes
Esposito et al. (80)	75	1.8 or 3	8 h/day for up to 21 days	Increased cell division, cell densities, COL II, and chondrogenesis in MSCs
Fitzsimmons (73)	15	1	A single 30 min exposure	Prevented increases in NO, cGMP, and increased DNA content in proliferation rates of chondrocytes
Meyer-Wagner et al. (69)	15	5	45 min every 8 h, 3x/day for 21 days	Increased GAG/DNA and improved chondrogenic differentiation via COL II in BM-MSCs
Parate et al. (81)	15	2	1 application for 10 min	Increased Sox-9, COL II, and aggrecan. Stimulated chondrogenesis via calcium homeostasis in MSCs
Varani et al. (82)	75	1.5	Continuously for 1 week	Upregulated A_2A_ and A_3_ ARs increasing anti-inflammatory properties in both chondrocytes and FLS

*PEMF, pulsed electromagnetic field; Hz, Hertz; mT, milliTesla; h, hour; d, day; NO, nitric oxide; BM-MSCs, bone marrow mesenchymal stromal cells; GAG, glycosaminoglycans; cGMP, cyclic guanosine monophosphate; COL, collagen; AR, adenosine receptor; FLS, fibroblast-like synoviocytes*.

## PEMF as an Alternative to Biologics in the Treatment of RA

The cytokine network in RA is complex and involves an interplay of both pro-inflammatory and anti-inflammatory cytokines. Regulating this cellular microenvironment is essential to maintaining healthy MSC phenotype. In RA, the macrophage-mediated inflammatory response is the main source of proinflammatory cytokines, including TNF-α, IL-1β, IL-6, C-X-C motif chemokine ligand 4 (CXCL4), and CXCL7 (83). While data from clinical trials show some efficacy using biologic drugs, the blockade of these cytokines does not fully control RA in all patients (84, 85). Interleukin-4 (IL-4) and−10 (IL-10) are pleiotropic cytokines considered to be promising modulators to control RA, as these regulatory mediators may have a direct inhibitory effect on the macrophage activity in the synovium (86, 87). While the targeted suppression of key inflammatory pathways involved in joint inflammation and destruction allows better disease control, it comes at the price of elevated infection risk, since blockade of these pathways can lead to broad immunosuppression (88, 89). In addition, these drugs are expensive, costing around $1,000–$3000 US per month, and the risks of prolonged treatment remain uncertain (87). While biologic drugs for RA work by halting the progression of joint damage, and sometimes pushing RA into remission, preliminary evidence shows loss of efficacy over time; therefore, rotation between available biological drugs is often necessary to maintain a good clinical response (89). Another unknown is the appropriate treatment duration for biologic medications. Once remission of the disease is achieved, it is unclear whether the drugs need to be maintained, or if they can safely be suspended (87, 90).

The pro-inflammatory transcription factor nuclear factor kappa B (NF-kB) plays crucial roles in the regulation of inflammation and immune responses by controlling the transcription of multiple cytokine genes (e.g., TNF-α, IL-1, IL-6, and INF-γ), as well as genes involved in cell survival. Given its central role in the control of inflammation and immunity, it is not surprising that inappropriate NF-kB activity has been linked to many autoimmune and inflammatory diseases, including RA (91–93). Exposure to PEMF induces early upregulation of adenosine receptors A_2A_ and A_3_ that reduce PGE_2_ and pro-inflammatory cytokines such as TNF-α, which combine to inhibit the activation of transcription factor NF-kB (94, 95). Specifically, at 5 Hz, 0.04 mT, a 1 h exposure to PEMF has been shown to down-regulate both NF-kB and TNF-α in murine macrophages (75). By inhibiting NF-kB activation (94), exposure to PEMF led to decreased production of TNF-α, IL-1β, IL-6, and PGE_2_ in human chondrocytes, osteoblasts, and synovial fibroblasts (94, 96).

It is important to note inflammatory cytokines can prevent MSCs differentiation, repressing their stem cell function. Cytokines, ions, growth factors, and chemokines modulate physiological processes of MSCs through their microenvironment (97). In both animal and clinical trials, TNF-α, IL-1β, IL-6, PGE_2_, and the anti-inflammatory cytokine IL-10 have all been shown to be modulated by PEMF (98–101). Exposure to PEMF has also been shown to stabilize plasma membrane Ca^2+^ ATPase (PMCA) activity (35). PMCA is a transport protein that removes Ca^2+^ from the cell, and thereby regulates the intracellular concentration of Ca^2+^ in all eukaryotic cells (102). These extremely low frequencies have a documented record of long-term safety, and their anti-inflammatory properties are well-established in animal arthritis models (35, 37). In double-blind clinical trials in which the knees and spine of RA patients were exposed to 5 Hz, 10–20 Gauss PEMF exposure for 10–30 min/day, 3–5x/ week for 1 month, up to a 47% improvement was documented in various clinical measures such as pain severity, joint tenderness and range of motion (24, 103). These beneficial clinical effects were attributed to PEMF's ability to significantly reduce the production of the RA-associated inflammatory cytokines IL-1β, IL-6, TNF-α, and PGE_2_, while increasing the levels of the anti-inflammatory cytokine IL-10 in peripheral blood mononuclear cells (PBMCs) such as T-cells and macrophages (26, 96, 104).

Table 2 provides a summary of the various parameters with which PEMF has been explored to-date for its ability to modulate cytokines and growth factors.

**Table 2:** Frequency Specific Effects of PEMF on cytokines and growth factors associated with RA.

**Authors**	**Frequency (Hz)**	**Field strength (mT)**	**Time of exposure**	**Outcomes (*in vitro*?)**
Gomez-Ochoa et al. (26)	50/60	15	15 min/day/days 7, 8, 9	Significantly decreased IL-1β and TNF-α, while increasing IL-10 in human fibroblasts
Ongaro et al. (96)	75	1.5	24 h	Inhibited release of PGE_2_, and IL-1β and IL-6 production, while stimulating release of IL-10 in synovial fibroblasts
Ross and Harrison (75)	5.1	0.04	1 h	Inhibited production of TNF-α and NF-kB in macrophages
Tang et al. (105)	15	1	6 h	Significantly decreased production of IL-1α and IL-6 in vertebral joint cells
Vincenzi et al. (94)	75	1.5	24 h	Inhibited NF-kB activation, and decreased the production of IL-6 and PGE_2_ in chondrocytes

*PEMF, pulsed electromagnetic field; Hz, Hertz; mT, milliTesla; h, hour; TNF-α, tumor necrosis factor alpha; IL, interleukin; PGE_2_, prostaglandin E_2_; VEGF, vascular endothelial growth factor; NF-kB, nuclear factor kappa B*.

## Ability of ELF-PEMF to Potentially Restore Angiogenic Homeostasis

Angiogenesis is the formation of new capillaries from pre-existing vasculature, and this process plays a critical role in the pathogenesis of several inflammatory autoimmune diseases such as RA (106). In RA, excessive infiltration of circulating leukocytes into the inflamed joint induces synovial tissue macrophages and fibroblasts to produce inflammatory and proangiogenic factors, such as TNF-α, IL-1β, IL-6, IL-17, and TGF-β that trigger neoangiogenesis (95, 106, 107). This inappropriate neoangiogenesis is also known to play a key role in the abnormal tissue growth, disordered tissue perfusion, abnormal ossification, enhanced responses to normal or pathological stimuli (108), and the development of the hyperplasic proliferative pathologic synovium (7). This area, called “pannus,” destroys articular cartilage, subchondral bone, and periarticular soft tissue, further increasing the density of synovial blood vessels required to develop the hyperplasic and invasive nature of the RA synovium (41). Although these newly formed blood vessels deliver oxygen to the augmented inflammatory cell mass, the neovascular network is dysfunctional and thus fails to restore tissue oxygen homeostasis. As a result, the rheumatoid joint remains in a markedly hypoxic environment (109). Hypoxia has been shown to activate NF-kB, which in turn activates macrophages, fibroblasts, and endothelial cells (107), stimulating further release of proinflammatory cytokines and growth factors (110–112) that directly or indirectly mediate inflammatory angiogenesis (113, 114). Repetitive cycles of hypoxia and reoxygenation, together with oxidants produced by phagocytic cells, promote a state of chronic oxidative stress within the microenvironment of the affected joint, leading to the generation of reactive oxygen species (ROS), which can further contribute to tissue damage. Given the central role neoangiogenesis plays in the pathogenesis of RA, anti-angiogenic therapy appears ideal.

While angiogenesis forms from new capillaries from pre-existing vessels, vasculogenesis is established capillarity formation from endothelial precursor cells (EPCs). Current understanding of the role of angiogensis and vasculogensis in RA is a focus of therapeutic intervention (115). Angiogenesis is profuse in RA and causes defective EPC function, leading to atherosclerosis and vascular disease in arthritis (115). Angiogenesis is essential for the expansion of synovial tissue in RA: pre-existing vessels facilitate the entry of blood-derived leukocytes into the synovial sublining, to generate and potentiate inflammation. Several steps are involved in angiogenesis, each of which is modulated by specific factors (10). The process starts with growth factors such as vascular endothelial growth factor (VEGF) and fibroblast growth factor (FGF) binding to their cognate receptors on endothelial cells (ECs) and activation of these cells to produce proteolytic enzymes (116). Recent evidence has emerged that implicates VEGF to be one of the key players in RA pathogenesis and vascular abnormalities (7, 41). For example, VEGF expression levels in synovial fluid and tissues have been shown to correlate with the clinical severity of RA, and with the degree of joint destruction (117). Proangiogenic factors such as VEGF are modulators of change in vascular permeability, and studies suggest that capillaries are more deeply distributed in the RA synovium, compared with normal tissue (118, 119). The synthesis of VEGF is induced by cytokines and growth factors (e.g., TNF-α), and through oxidative stress, and hypoxia (117, 120). Overexpression of VEGF-C in FLS by stimulation with TNF-alpha may play an important role in the progression of synovial inflammation and hyperplasia in RA by contributing to local lymphangiogenesis and angiogenesis (121). Both oxidative stress and hypoxia are present within the joints of RA patients (117). TNF-α has also been reported to induce the release of VEGF from endothelial cells (122), which can lead to an imbalance between endothelial cells (EC) tube formation and the parallel development of MSCs/pericytes and thereby altering angiogenesis and vasculogenesis (107).

MSCs are perivascular cells that are precursors of pericytes and adventitial cells that envelop microvessels and surround larger arteries and veins, as well as the myriad of other stromal cells that act in concert to maintain/restore tissue homeostasis (123, 124). Aberrant MSCs can release various inflammatory cytokines and VEGF (85), enhancing tissue inflammation (108), and promoting angiogenesis, both of which are of direct relevance to the pathogenesis of RA (125). Pericytes have been shown to possess stem-like qualities, and have been hypothesized to be the *in vivo* counterparts, or precursors, of MSCs (126–128). MSC/pericytes are recognized for their central role in blood vessel formation, and they act as a repair system in response to injury by maintaining the structural integrity of blood vessels (129). Pericytes have been shown to both stabilize and promote capillary sprouting (130). Perivascular pericytes envelop the vascular tube surface of the inner EC layer that lines the blood vessel wall (131). Because of their close anatomical and functional association with ECs, pericytes are thought to regulate capillary diameter and physically influence EC behavior (132) via contraction in response to electrical or neurotransmitter stimulation (133). Homing of endothelial progenitor cells (EPCs) to an RA injury site is important for repair of vasculature and angiogenesis. Applied direct current (DC) electric fields has been reported to guide EPC migration through VEGF receptor signaling *in vitro*, controlling EPC behavior to heal injury sites in the vascular (134). PEMF has also been reported to increase the number and function of circulating EPCs in treating myocardial ischemia/reperfusion (I/R) injury in rats (135).

Collectively, these data point to EPCs and MSCs as highly localized modulators of blood flow (130). It has also been found that MSCs can stabilize blood vessels and contribute to tissue and immune system homeostasis under physiological conditions by assuming a more active role in tissue repair in response to injury (136). As such, MSCs/pericytes represent a logical target for new *in vivo* therapeutic approaches to treating the vascular abnormalities present in RA and halting disease progression to restore homeostasis (136). Since PEMF have been shown to stimulate the production of MSCs (137), and MSCs can stabilize blood vessels and contribute to immune system homeostasis, the possibility exists that PEMF could provide a therapeutic application to restore immune balance and bringing hypoxic conditions and synovial angiogenesis back to a state of homeostasis.

**Figure 1:**
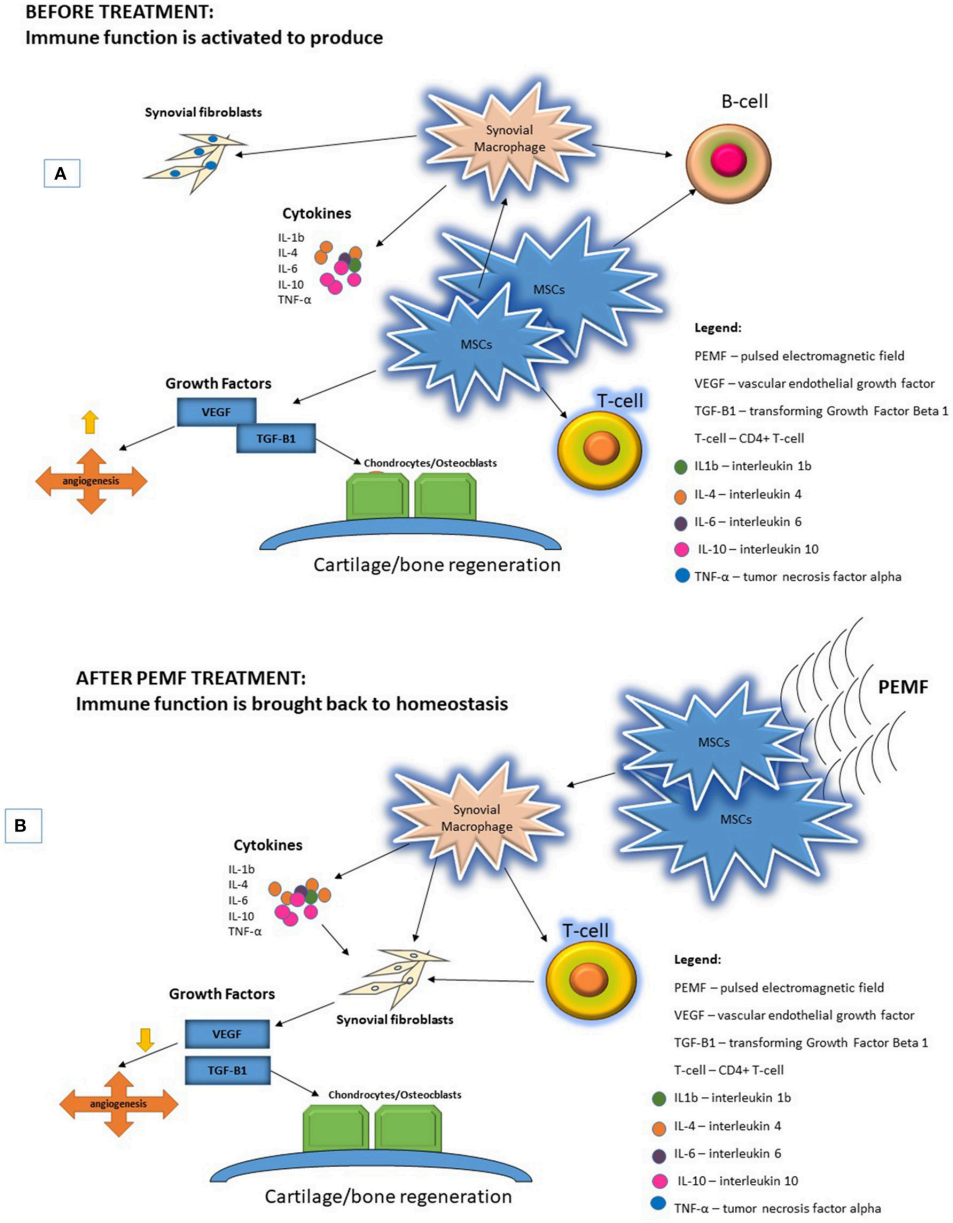
PEMF are physical stimuli that produce membrane activations of multiple cellular pathways. **(A)** RA pathogenesis begins with activation of immune function increasing proinflammatory cytokines and upregulating growth factors to increase FLS proliferation and bone resorption. **(B)** Application of PEMF could potentially bring immune function back to homeostasis.

MSCs represent an ideal target on which PEMF can initiate their effects on the aberrant immune response that drives the pathogenesis of RA. MSCs/pericytes down-modulate the production of synovial macrophages, which trigger production of cytokines, such as IL-4, that initiate the proliferation of synovial fibroblasts, promoting the expression of growth factors such as VEGF and TGF-β (138, 139). Exposure of MSCs/pericytes to PEMF appears to trigger a cascade of downstream effects on multiple pathways, affecting macrophages, T-cells, and B cells, and the cytokines that are produced. The cumulative result of these varied effects is modulation of VEGF and TGF-β, which ultimately curtails the production of synovial fibroblasts and osteoclasts and halts bone resorption, while promoting the production of chondrocytes and osteoblasts to restore cartilage and bone health/integrity (Figure 1).

The effects of PEMF on vessel growth and development, both *in vitro* and *in vivo*, support the use of this approach to therapeutically modulate the aberrant angiogenesis present in RA, (140–142). PEMF has been reported to improve osteochondral ossification, and modulate nociception (143–146) through the down-regulation of neovascularization (15, 147, 148) in both animals and humans with RA (9, 24, 25, 27, 149). It has also been reported to significantly reduce activation levels of VEGF (15), to inhibit the proliferative ability of human umbilical vein endothelial cells (HUVECs) (148), and to reduce the extent of vascularization in diseased tissue (142). Approximately half of the cited studies of PEMF application indicate a vasodilatory effect, the magnitude of which is dependent upon the initial vessel tone. The remaining half indicates that PEMF has the potential to trigger vasoconstriction. The ultimate outcome of PEMF application thus appears to depend on the cellular/mechanistic basis of the disease in question (140). A summary of some of the studies that have explored the use of various regimens of PEMF to potentially restore angiogenic homeostasis appear in Table 3.

**Table 3:** Frequency Specific Effects of PEMF on angiogenesis-associated RA.

**Authors**	**Frequency (Hz)**	**Field strength (mT)**	**Time of exposure**	**Outcome**
Delle-Monache et al. (15)	50	2	1, 6, and 12 h	Significantly reduced the expression and activation levels of VEGF in HUVECs
Leoci et al. (150)	8	1.05	5 min/2x/day for 3 weeks	Reduction in peak gradient blood flow in prostatic hyperplasia
Okana et al. (141)	Static	120	24/7 for 10 days	Significantly promoted tubular formation in area density and length of tubules and improved gradient force on vessels
Vincenzi et al. (94)	75	1.5	24 h	Inhibited VEGF activation in chondrocytes
Wang et al. (148)	Static	2–4	24 h	Significantly inhibited the proliferation ability of HUVECs to treat pathological angiogenesis

*PEMF, pulsed electromagnetic field; Hz, Hertz; mT, milliTesla; HUVEC, human umbilical vein endothelial cell; VEGF, vascular endothelial growth factor; ECs, endothelial cells*.

## Conclusion

Under normal physiological conditions, MSCs in the joint are believed to contribute to the maintenance and repair of joint tissues. In RA, however, the repair function of MSCs appears to be repressed by the inflammatory milieu. In addition to being passive targets, MSCs could interact with the immune system and play an active role in the perpetuation of arthritis and progression of joint damage (54). Achieving homeostasis in the face of acute inflammatory/immune challenges in the human body involves maintaining a balance of highly complex biochemical and cellular interactions. When this delicate balance is upset, acute inflammatory and immune responses designed to quickly eliminate a transient threat become chronic, and inflammatory/autoimmune disease sets in. RA is a paradigmatic autoimmune disease, and current RA therapies target inflammatory molecules involved in autoimmune activation. Despite the therapeutic improvements in RA, there are still a substantial number of patients who respond only transiently to these approaches, and others who do not respond at all. As such, there is an urgent unmet need to identify complementary and innovative therapies for the treatment of RA.

PEMF is emerging as a novel and highly promising means of treating chronic inflammation and aberrant immunity that exists in diseases such as RA. It can be used to target aberrant MSCs to potentially bring the inflammatory milieu back to homeostasis. Cellular electrical properties such as membrane surface charge and membrane potential can be readily influenced by PEMF (151–153), which can affect oscillatory frequencies of the myriad of enzymes present within the cells. PEMF can also influence cell membranes, nucleic acids, and bioelectrical phenomena generated by coherent groups of cells that are essential to cell-to-cell communication processes (154, 155). PEMF appears to exert its effects on cellular function and differentiation by altering the spatial and temporal patterns of intracellular calcium (Ca^2+^) concentration (10) and restoring levels/activity of potassium (K^+^) channels (17, 156, 157). By restoring normal Ca^2+^ ion flux and Na+/K+ balance, the cell can begin the process of down-regulating inflammatory cytokines, heat-shock proteins, and proangiogenic molecules such as VEGF (157), making it possible for the body to commence rebuilding healthy cartilage. Using PEMF to modulate inflammation and immune function is relatively safe in contrast to the broad immunosuppression currently in clinical favor (39, 158). An alternative to immunosuppression–healthy immunomodulation and tissue repair–can be achieved by targeting MSCs with PEMF. While traditional approaches target individual molecules or signaling pathways, PEMF works on all cellular/organismal systems in a holistic and integrative manner by potentially bringing the transmission and flow of information (signal transduction) back to a state of homeostasis via coherence of sinusoidal pulses (159). There are other potential advantages of PEMF including low-cost, easy-to-use at-home, without adverse effects. While cell therapies or biologics suffer from the possibility of loss of efficacy over time (87), preliminary clinical studies with PEMF have shown no loss of efficacy even after exposure to the field has ended (160). Another key unsolved problem in the treatment/management of RA is determining the optimal duration of therapy, and the lack of data to inform clinicians whether drugs should be suspended once remission of the disease is obtained (87). PEMF has the advantage of use without concerns regarding global immunosuppression until the desired clinical outcome is obtained (87). Since MSCs are ubiquitous, targeting their regenerative, and anti-inflammatory capacities would be an optimal combination of exogenous (PEMF), and endogenous (MSC) therapies. Clinical applications include whole-body mats for systemic approach (161), and hand-held devices for localized therapy (149). For localized applications, direct capacitive coupling mechanisms such as electrodes adhere to the site of inflammation/tissue degeneration. For non-direct capacitive/inductive coupling, mats can be used for full body applications. Current research shows optimal frequency < 75 Hz, with optimal intensity (field strength) < 5 mT, and optimal time courses ranging between 15 and 90 min, with longer duration most effective for severe symptoms.

## Author Contributions

GA-P provided expertise and contributed editorial and written content on mesenchymal stromal cells (MSCs). DA provided expertise on RA and contributed editorial and written content on RA pathology. CR wrote the manuscript and provided expertise on the therapeutic effects of pulsed electromagnetic field for the treatment of RA.

### Conflict of Interest Statement

The authors declare that the research was conducted in the absence of any commercial or financial relationships that could be construed as a potential conflict of interest.
